# A novel study on the quality of life index in canine chronic kidney disease treated with incremental intermittent hemodialysis

**DOI:** 10.14202/vetworld.2024.1702-1714

**Published:** 2024-08-04

**Authors:** Akashpreet Singh, Randhir Singh, Dhiraj Kumar Gupta, Raj Sukhbir Singh

**Affiliations:** 1 Department of Veterinary Medicine, Guru Angad Dev Veterinary and Animal Sciences University, Ludhiana - 141 004, Punjab, India; 2 Department of Teaching Veterinary Clinical Complex, Guru Angad Dev Veterinary and Animal Sciences University, Ludhiana - 141 004, Punjab, India

**Keywords:** chronic kidney disease, dialysis adequacy, incremental intermittent hemodialysis, quality of life index

## Abstract

**Background and Aim:**

In veterinary medicine, health-related quality-of-life index (QOL*i*) measurements are becoming increasingly important because they are a multifaceted concept that represents not only patients’ physical well-being but also clients’ emotional health. This study assessed QOL*i* in dogs receiving incremental intermittent hemodialysis (*i*-IHD) with high- and low-flux dialyzers.

**Materials and Methods:**

Thirty dogs diagnosed with chronic kidney disease (CKD) Stage IV were randomly divided into two groups of 15 dogs each. A high-flux dialyzer was used in Group I, whereas a low-flux dialyzer was used in Group II. *i*-IHD was performed on days 0, 2, 4, 19, and 34, whereas QOL*i* evaluation was performed on days 0, 15, 30, and 45.

**Results:**

Both groups exhibited considerable decreases in post-dialysis creatinine, blood urea nitrogen, and phosphorus levels, while Group I experienced notable reductions in post-dialysis triglyceride and cholesterol levels. Dialysis adequacy did not show any significant difference between the clearance rates of high- and low-flux dialyzers. The QOL*i* assessment showed better post-dialysis scores in all categories except for water balance in Group I, while Group II demonstrated a worsening trend in scores for mental status, appetite, mobility, general health, and pain.

**Conclusion:**

In the first three sessions of *i*-IHD, dogs with CKD should be treated every other day, and the schedule can be extended by 15 days after that. A high-flux membrane, which effectively decreases triglyceride and cholesterol levels more than a low-flux membrane, warrants consideration for dogs with cardiovascular complications undergoing dialysis. The dialysis-related QOL*i* aids in clinical decision-making and encourages client engagement.

## Introduction

Chronic kidney disease (CKD) in dogs reduces overall health-related quality of life index (QOL*i*) due to functional limitations in various daily activities. Assessing a pet’s (QOL*i*), which encompasses physical and mental well-being, is gaining significance in veterinary medicine for those with chronic conditions [1]. Evaluating the quality of care and determining the efficacy of veterinary medical interventions enhance clinical decision-making. Besides evaluating patient progress, clinicians can measure the impact of certain conditions and therapies using QOL*i* scores [2]. Renal failure in dogs has become a complex condition where the kidney significantly contributes to the development of multi-organ failure [3]. CKD is defined as irreversible structural and/or functional impairment of one or both kidneys that persists for more than 3 months and is stable for a time, and eventually progresses to end-stage kidney disease. Dogs with Stage III and IV CKD can have moderate-to-severe azotemia, with serum creatinine levels of 2.9–5.0 mg/dL and above 5.0 mg/dL, respectively, and the clinical symptoms are more pronounced, necessitating close supervision and patient treatment. The diagnosis of renal failure is typically based on a combination of a complete patient history, physical examination, laboratory data, and imaging, all of which play an important role in distinguishing acute from chronic renal diseases [4]. CKD is also a major secondary disorder, leading to systemic hypertension in dogs. According to International Renal Interest Society (IRIS) staging system for CKD (Modified, 2023), systolic blood pressure (SBP) ≥160 mmHg warrants treatment with an antihypertensive drug(s) [5].

Intermittent hemodialysis (IHD) is a technically sophisticated treatment modality for removal of uremic toxins and correction of fluid and electrolyte imbalances [6]. Indication for IHD in dogs is a life-altering process that requires decision-making and financial and emotional investment on part of the client to ensure the desired outcome. IHD can be performed using either high-flux or low-flux membranes. Compared with low-flux membranes, high-flux membranes have a larger pore size, which allows the diffusion of a greater number of uremic toxins and middle-sized molecules such as ß_2_ microglobulin and p-cresol. Similar to their human counterparts, dogs with CKD were initially treated with a conventional regimen of 3 days a week IHD on alternate days. To reduce the frequency of dialysis, the new concept of incremental IHD (*i*-IHD) is gaining popularity among human nephrologists [7]. *i*-IHD involves tailoring the initial hemodialysis prescription to a dose <3 times weekly or reducing the dialysis dose in patients with significant residual kidney function [8]. To date, no such study has been undertaken in veterinary medicine, whereafter, after initial patient stabilization with a thrice-weekly dialysis regimen, subsequent dialysis sessions can be performed depending on the residual kidney function.

Considering the above points, the current study aimed to assess the QOL*i* and effectiveness of high- and low-flux membranes in dogs with Stage IV CKD by applying the idea of *i*-IHD.

## Materials and Methods

### Ethical approval and Informed consent

All experimental procedures were performed in accordance with the university’s institutional animal ethics committee (IAEC) rules and regulations. The study was approved during the 62^nd^ meeting of the IAEC (vide letter No. GADVASU/IAEC/2021/294-315, dated October 27, 2021).

Before inclusion, all clients were thoroughly informed and counseled about their financial, emotional, and time investment. The study period involved 45 days, covering five *i*-IHD sessions on days 0, 2, 4, 19, and 34, and quality of life evaluation up to day 45. The dialysis cost for the first three sessions was borne by the clients (INR 3,500 or USD 42.07 per session), and the cost of the remaining two sessions was covered by the institution.

### Study period and location

The study was conducted from October 2021 to October 2022 at Dialysis Unit, Multi-specialty Veterinary Hospital (MSVH), Guru Angad Dev Veterinary and Animal Sciences University, Ludhiana, Punjab, India.

### Study population and patient characteristics

This prospective study involved 30 client-owned dogs of both sexes and varying breeds diagnosed with stage IV CKD, according to IRIS modified guidelines (IRIS, 2019).

The selection criteria for all the dogs with IRIS CKD Stage IV were based on a detailed history, hemato-biochemical evaluation, routine urine analysis, blood pressure monitoring, and imaging findings. Dogs were included if they had two or more clinical signs associated with CKD for at least 3 months. The clinical signs considered were polyuria, polydipsia, halitosis, melena, vomiting, weight loss, and anorexia. All studied dogs had serum creatinine levels of >5 mg/dL and had undergone a minimum of 1 week of conventional medicinal therapy (parenteral fluids, diuretics, and anti-emetics) for the management of CKD without any favorable response. Further, the diagnosis was supported by ultrasound findings of kidneys consistent with CKD.

Dogs with a vague history or those not having a combination of two minimum clinical symptoms of CKD from the past 3 months, acute-on-CKD, shock, sepsis, and any other concurrent primary infectious, neoplastic, hepatic, or vector-borne diseases were excluded from this study. On the day of presentation, all patients were thoroughly evaluated for age, sex, body weight, body condition score, and geographic area. Each dog was evaluated for body vitals like rectal temperature, heart rate, and respiratory rate 15 min after complete rest on arrival. Furthermore, urinalysis, radiography, ultrasonography, and electrocardiogram (ECG) were only performed once on day 0 to aid in the proper diagnosis.

After confirming Stage IV CKD, the dogs were randomly divided into two groups, each with 15 dogs.

- Group I (n = 15): *i*-IHD with high-flux membrane
- Group II (n = 15): *i*-IHD with low-flux membrane

### Urinalysis

Urine samples were collected in a sterile plastic container through cystocentesis and examined for color, smell, turbidity, and sediment, followed by microscopic examination of casts, cells, and crystals. Urine specific gravity (USG) was measured using a hand-held refractometer (Master Refractometer, Atago, Japan). The device was standardized using distilled water to 1.000 reference value. A urine drop was placed on the stage, and the value was noted. In addition, urine samples were qualitatively analyzed for ketone bodies, nitrite, protein, glucose, blood, leukocytes, urobilinogen, bilirubin, and pH using an SIEMENS Multistix 10 SG (Siemens Healthcare Pvt. Ltd., India). The readings were noted on Orinalyser U 120 analyzer (Arkray Health Pvt. Ltd., India).

### Radiography

All dogs were subjected to X-ray examination of the thorax and abdomen (in lateral and ventrodorsal view) to rule out concurrent abnormalities such as cardiomegaly, pleural effusions, mass, urolith, etc., and to measure kidney size with respect to the second lumbar vertebrae (L_2_).

### Abdominal ultrasound

All dogs were subjected to abdominal ultrasonography using the B mode of a Philips Affinity 70 ultrasound machine (Philips India Ltd.). Ultrasonography findings related to the kidneys, ureters, urinary bladder, intestines, liver, spleen, prostrate, or uterus were noted.

### Blood pressure monitoring and treatment protocol for hypertensive dogs

SBP was monitored during both the pre- and post-dialysis sessions on days 0, 2, 4, 19, and 34 using a Doppler unit, sphygmomanometer, ultrasound coupling gel, and inflatable cuff (Doppler vet BP mano medical, France). For SBP measurement, each dog was placed in the left lateral recumbency position, and once the artery was located, the probe was held still and the cuff was inflated until the “whooshing” sound of the pulse was no longer heard. Gradually, the cuff was deflated using the pressure-release button/valve and the participant was listened for the return of the “whooshing” sound to evaluate SBP. Seven readings were taken, and the average was used to determine the final SBP. Dogs with SBP of 160–179 mmHg were treated with angiotensin-converting enzyme inhibitor (ACEI) enalapril (Tablet Envas, Cadila Pharmaceuticals Ltd., India) at 0.5 mg/kg body weight orally once daily. In contrast, dogs with SBP ≥180 mmHg were managed with a combination of ACEI enalapril and calcium channel blocker (CCB) amlodipine (Tablet Amlokind 5, Mankind Pharma Ltd., India) at 0.5 mg/kg body weight orally once daily.

### Electrocardiography

ECG data were recorded using Bailey’s hexanal limb leads I, II, III, augmented vector right (aVR), augmented vector left (aVL), and augmented vector foot (aVF) using a Cardiart 8108 British Physical Laboratories (BPL) six-channel electrocardiographic machine (BPL Medical Technologies, Bangalore, India). Lead II was used to interpret ECG readings. ECG readings were obtained at a speed of 50 mm/s with the help of various leads. During catheterization and ongoing dialysis, ECG was monitored using a multiparameter monitor (Philips Efficia CM120, China).

### Hematological evaluation

All dogs were evaluated at pre- and post-dialysis sessions for complete blood count, which included hemoglobin (Hb), total leukocyte count, differential leukocyte count (DLC), packed cell volume (PCV), and platelet count (PLT). In addition, blood smears of all dogs were examined microscopically for commonly prevalent hemoparasites causing Babesiosis, Anaplasmosis, and Ehrlichiosis.

### Biochemical analysis

A fully automatic chemistry analyzer (Ortho Clinical Diagnostics, Johnson & Johnson, Co., USA) was used for the analysis of the following biochemical parameters using Vitrose DT slides:

#### Renal function parameters

Serum creatinine (Cr), blood urea nitrogen (BUN), sodium (Na), potassium (K), chloride (Cl), calcium (Ca), and phosphorous (P).

#### Liver function parameters

Alanine transaminase, aspartate aminotransferase, gamma-glutamyl transferase, alkaline phosphatase, total bilirubin, total protein (TP), albumin, blood glucose, cholesterol, and triglycerides.

### *i*-IHD and dialysis adequacy

**Figure-1:**
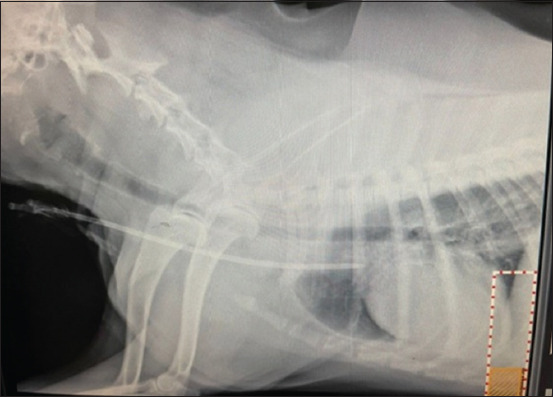
Lateral radiograph of dog with proper positioning of the catheter tip at the junction of the cranial vena cava and right atrium.

The procedure was performed using a Fresenius 4008S machine (Fresenius Medical Care, Germany). The dialyzer models used were also of Fresenius medical care, that is, high-flux (FX 60) and low-flux (FX 8) models with surfaces of 1.4 square meters in both membranes. A dedicated double-lumen dialysis catheter was used, which allows the simultaneous removal and return of blood. We used temporary catheters made of polyurethane with a diameter and length of 11.5 Fr × 19 cm (ST. Stone Medical Devices Private Limited, India). After catheterization, the dogs were again subjected to thoracic radiography in lateral view to determine the proper positioning of the catheter tip at the junction of the cranial vena cava and right atrium (Figure-1). Initially, to achieve patient stabilization, the first three dialysis sessions were conducted on alternate days (day 0, 2, and 4), followed by an increment of 15 days for subsequent sessions (day 19 and 34). The blood pump speed and dialysis session time were kept constant in both membrane groups, i.e., session I @ 2 mL/kg/min for 2 h; session II @ 5 mL/kg/min for 4 h; and sessions III, IV and V @ 7 mL/kg/min for 6 h. The adequacy of dialysis sessions was evaluated using fractional urea clearance (Kt/V), urea reduction ratio (URR), and creatinine reduction ratio (CrRR). The above parameters were calculated using an online calculator (www.omnicalculator.com). The formulas are as follows:

Kt/V = ln [(Post BUN/Pre BUN) - (0.008 × Dialysis duration) + (4–3.5 × (Post BUN/Pre BUN)] × (UF/Weight)

Where, 1n stands for the logarithm with the base of e, the natural logarithm; Post-BUN, post-dialysis BUN; Pre-BUN, pre-dialysis BUN; ultrafiltration (UF), the volume of removed ultrafiltrate; and weight, the post-dialysis weight of dog in kilograms.

URR = (U_pre_ - U_post_)/U_pre_ × 100 = (1 - U_pre_/U_post_) × 100

Here, U_pre_ denotes pre-dialysis BUN, and U_post_ denotes post-dialysis BUN.

CrRR = (Cr_pre_ - Cr_post_)/C_pre_ × 100 = (1 - Cr_pre_/Cr_post_) × 100

Here, Cr_pre_ denotes pre-dialysis creatinine, and Cr_post_ denotes post-dialysis creatinine.

The adequacy of dialysis was checked according to the treatment intensity prescription recommendations for dogs [9]. During non-dialysis days, dogs were prescribed renal diet (Kidney care, Hill’s prescription diet, USA), calcium acetate at 60–90 mg/kg/day orally thrice a day (Tablet Phostat, Zydus Healthcare Ltd., India), omega-3 and 6 fatty acids at 2.5 mL/10 kg PO BID (Syrup FO-120, Skooner Pharma, India), L-carnitine at 50 mg/kg orally once a day (Tablet Carnisure, Torrent Pharmaceuticals Ltd, India), gut pre-and pro-biotics at 2 g/dog orally twice a day (Sachet GR-180, Skooner Pharma), and anti-fibrotics at 1 mL/5 kg orally twice a day (Syrup Heckler Pro, Skooner Pharma).

### QOL*i*

A renal disease/dialysis-oriented QOL*i* questionnaire was prepared, which was filled by the clients before the start of the first dialysis session, that is, on day 0, and then on days 15, 30, and 45 to observe the QOL after hemodialysis in all the studied dogs. The QOL*i* questionnaire assessed various aspects related to renal failure and dialysis. The parameters included happiness, mental status, pain, appetite, hygiene, water balance, mobility, general health, quality of life assessment, and quality of life of the client. Before filling in the questionnaire, all clients were thoroughly sensitized about its significance, and the same person was employed to answer the questionnaire throughout the study period. Furthermore, to avoid inconvenience to clients for filling out the questionnaire on non-dialysis days, the QOL*i* questionnaire on day 0 was completed by the client in person. In contrast, subsequent assessment was carried out telephonically involving the same client and research scholar.

### Statistical analysis

The data were analyzed using SAS statistical software version 9.2 (SAS Institute Inc. USA). Data are presented as mean, standard error of mean (SE), minimum-maximum values, frequency, and proportion. The paired t-test was used to examine the significance of differences between pre- and post-treatment values of hemato-biochemical parameters for each membrane type group. The two-sample t-test was employed to examine the significance of differences between blood pressure values in the high-flux and low-flux membrane groups. Further, Mann–Whitney test was used to compare QOL*i* between the high- and low-flux membrane groups. Kruskal–Wallis test was used to observe significant differences over the study period for various QOL parameters within the high- and low-flux membrane groups. The level of significance was set at level p < 0.05.

## Results

### Study population and patient characteristics

Approximately 20% and 40% of the dogs were below 3 years of age in high- and low-flux groups, respectively. Eighty percent of the dogs in the high-flux group were older than 6 years of age (6.1–8 years: 40% and >8.1 years; 40%), whereas approximately 60% of the dogs in the low-flux group were older than 6 years of age (6.1–8 years; 33.3% and >8.1 years: 26.7%). The majority of dogs were male in both groups (93.3%). The average body weight (kg) of dogs in groups I and II was 32.77 ± 1.97 and 27.52 ± 3.04, respectively. Dogs in both groups had mean body condition scores of 3.13 ± 0.19 (Group I) and 3.10 ± 0.24 (Group II) on a 5-point scale. The majority of dogs in both groups (66.6% and 80%) were from urban areas. All body vitals (temperature, respiratory rate, and heart rate) on the day of presentation were within normal physiological ranges.

### Routine urinalysis

One dog in each group had a urine protein level of 1+. Seven (46.7%) and 04 (26.7%) dogs had urine protein of 2+ in the high- and low-flux groups, respectively. The remaining dogs in both groups had urine protein levels of 3+. The USG was 1.015–1.025 in 66.7% and 60% of dogs in Groups I and II, respectively. The remaining dogs in both groups had USG of <1.015. Microscopic examination of urine samples from both groups revealed waxy casts, granular casts, and struvite crystals.

### Radiographic findings

**Figure-2:**
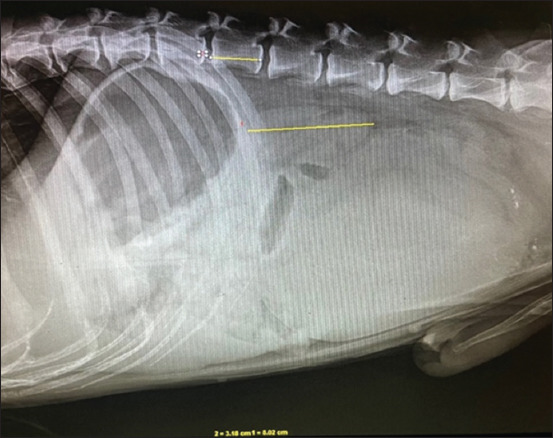
Lateral radiograph of dog’s abdomen to evaluate kidney size with respect to 2^nd^ Lumbar vertebrae (L_2_).

The kidneys appeared smaller than the L_2_ vertebrae in 60% and 66.7% of dogs in Groups I and II, respectively (Figure-2). No other radiographic abnormalities were detected in the abdomen or thorax.

### Ultrasound findings

**Figure-3:**
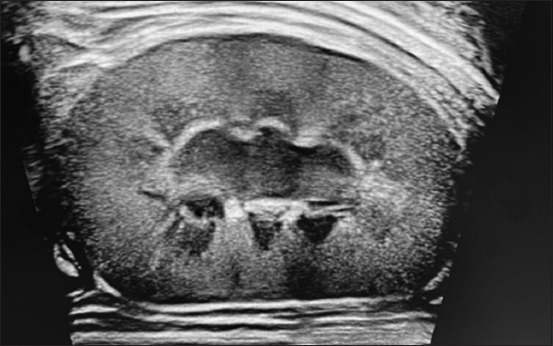
Abdominal ultrasound revealing hyperechoic renal cortex.

**Figure-4:**
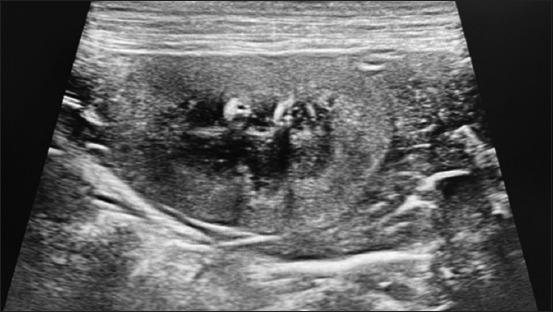
Abdominal ultrasound of kidney revealing complete loss of cortico-medullary differentiation.

**Figure-5:**
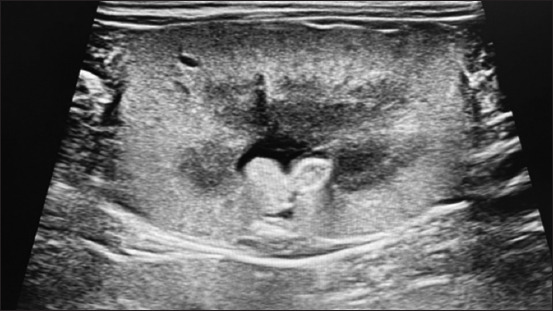
Abdominal ultrasound revealing renal cyst.

Renal ultrasound revealed hyperechoic cortex in all dogs, with poor corticomedullary differentiation (60%) in Group I and 66.7% in Group II. Two dogs in the high-flux group had renal cysts, and one dog had a nephrolith (Figures-3–5).

### Electrocardiography

No ECG abnormalities were detected in any of the dogs on presentation. During catheterization, transient arrhythmia was evident when the guide wire tip touched the right atrium.

### Blood pressure monitoring and treatment

In the high-flux group, 33.3% of the dogs were normotensive (SBP <140 mmHg), 13.4% were pre-hypertensive (SBP 140–159 mmHg), 20% were hypertensive (SBP 160–179 mmHg), and 33.4% were severely hypertensive (SBP ≥180 mmHg). Likewise, in the low-flux group, 40% of the dogs were normotensive, 20% were pre-hypertensive, 6.7% were hypertensive, and 33.3% were severely hypertensive. The maximum SBP recorded before the start of dialysis during session I was 220 and 270 mmHg in the high- and low-flux groups, respectively (Table-1). Within each group, a significant decrease (p < 0.05) in SBP was observed after dialysis during sessions I, II, and IV. Similarly, over the study period, the SBP reduced significantly (p < 0.05) from 167.53 ± 6.15 (Pre-dialysis; Session I) to 148.90 ± 11.4 (Post-dialysis; Session V) in the high-flux group and from 162.5 ± 2.95 (Pre-dialysis; Session I) to 143.6 ± 4.4 (Post-dialysis; Session V) in the low-flux group.

**Table-1:** Pre- and post-*i*-IHD systolic blood pressure (Mean ± SE) of dogs in high-flux (n = 15) and low-flux (n = 15) groups.

Session (Day)	Group	Systolic blood pressure		p-value

		Pre-dialysis	Post-dialysis	
I (Day 0)	High-flux	167.53 ± 46.15 (140–220)*	164.67 ± 5.89 (135–210)	0.015
	Low-flux	162.5 ± 11.4 (80–270)	158.6 ± 11.1 (90–265)	0.006
II (Day 2)	High-flux	166.87 ± 6.16 (138–220)	162.40 ± 5.96 (135–215)	0.002
	Low-flux	163.1 ± 11.3 (85–275)	157.1 ± 10.8 (78–260)	0.000
III (Day 4)	High-flux	155.27 ± 3.47 (135–180)	154.73 ± 4.21 (135–185)	0.735
	Low-flux	144.20 ± 6.81 (82–200)	141.87 ± 7.39 (78–210)	0.172
IV (Day 19)	High-flux	152.60 ± 3.88 (135–190)	149.73 ± 3.80 (135–189)	0.044
	Low-flux	143.20 ± 6.33 (80–190)	139.80 ± 6.47 (85–200)	0.029
V (Day 34)	High-flux	152.27 ± 3.51 (135–188)	148.40 ± 2.95 (138–182)	0.082
	Low-flux	143.27 ± 4.54 (100–180)	143.60 ± 4.42 (95–175)	0.850

* Values in parentheses indicate minimum and maximum values, *i*-IHD=Incremental intermittent hemodialysis, SE=Standard error

### Hematology

During the session, I, significant leukocytosis with absolute neutrophilia and thrombocytopenia was observed in the high-flux group during post-dialysis, whereas, in the low-flux group, only significant thrombocytopenia was observed during post-dialysis (Table-2). There was significant leukocytosis with absolute neutrophilia after dialysis in both membrane groups during session II. During session III, significant reductions in post-dialysis Hb and PCV were observed in the low-flux group. During session IV, a significant drop in post-dialysis Hb, total erythrocyte count (TEC), and platelets were observed within the low-flux group, along with a significant reduction in post-dialysis platelet levels was observed in the high-flux group. Significant leukocytosis along with absolute neutrophilia and reduced PLT during post-dialysis in the high-flux group was present during session V.

**Table-2:** Pre- and post-*i*-IHD hematological findings (Mean ± SE) of dogs in high-flux (n = 15) and low-flux (n = 15) groups.

Parameter	Dialysis	Session I		Session II		Session III		Session IV		Session V	
				
		High-flux	Low-flux	High-flux	Low-flux	High-flux	Low-flux	High-flux	Low-flux	High-flux	Low-flux
Hb (g/dL)	Pre	9.69 ± 0.92	7.82 ± 0.65	8.61 ± 0.85	8.78 ± 0.56	8.17 ± 0.63	8.56 ± 0.66^a^	8.79 ± 0.47	7.85 ± 0.50^a^	9.11 ± 0.38	8.04 ± 0.42
	Post	9.42 ± 0.80	7.39 ± 0.61	8.61 ± 0.91	8.1 ± 0.55	8.53 ± 0.65	7.89 ± 0.6^b^	8.68 ± 0.38	7.58 ± 0.46^b^	8.90 ± 0.36	8.03 ± 0.39
TLC (×10^3^/μL)	Pre	14337 ± 1869^a^	11440 ± 1028	15914 ± 1606^a^	12640 ± 1386^a^	14407 ± 1413	12827 ± 966	11927 ± 1205^a^	8953 ± 435	10460 ± 448^a^	10873 ± 366
	Post	17100 ± 2749^b^	12380 ± 1026	18703 ± 1815^b^	15033 ± 1423^b^	17053 ± 2414	12893 ± 1011	13153 ± 1424^b^	10047 ± 648	11273 ± 466^b^	11453 ± 533
Absolute neutrophils	Pre	12746 ± 1850^a^	10112 ± 1041	13884 ± 1378^a^	11479 ± 1374^a^	12770 ± 1387	11559 ± 1015	10524 ± 1191^a^	7924 ± 479^a^	9086 ± 411^a^	9785 ± 373
	Post	15618 ± 2640^b^	11134 ± 975	17043 ± 1696^b^	13997 ± 1404^b^	15450 ± 2331	11463 ± 932	11637 ± 1254^b^	9028 ± 62^b^	9895 ± 44^b^	10155 ± 465
Absolute lymphocytes	Pre	1508 ± 215	1275 ± 188	1876 ± 375^A^	1025 ± 128^B^	1470 ± 212^A^	2179 ± 986^B^	1344 ± 170	950.7 ± 82.1	1330 ± 180	1078 ± 108
	Post	1399 ± 260	1024 ± 220	1244 ± 231	976 ± 171	1499 ± 200	1375 ± 227	1736 ± 313	1002 ± 96.9	1378 ± 134	1298 ± 110
Absolute eosinophils	Pre	140.1 ± 51.3	63.5 ± 38.8	88 ± 44.2	168 ± 59.1	116.4 ± 47.2	54.5 ± 30.9	58.2 ± 50.7	78.4 ± 41.9	44.0 ± 35.5	10.1 ± 10.1
	Post	82.4 ± 56.7	104.3 ± 75.8	19.5 ± 19.5	65.1 ± 36.3	129.1 ± 86.9	55.2 ± 37.9	19.5 ± 19.5	53.2 ± 39.2	15.5 ± 12.5	19.5 ± 19.5
TEC (×10^6^/μL)	Pre	4.16 ± 0.42	3.42 ± 0.33	3.64 ± 0.36	3.75 ± 0.26	3.48 ± 0.31^a^	3.67 ± 0.26^a^	3.85 ± 0.26	3.62 ± 0.23^a^	4.42 ± 0.26	3.86 ± 0.38
	Post	3.10 ± 0.32	3.25 ± 0.28	3.56 ± 0.36	3.59 ± 0.23	3.71 ± 0.17^b^	9.39 ± 0.24^b^	3.93 ± 0.30	3.36 ± 0.23^b^	4.53 ± 0.25	3.90 ± 0.32
PCV (%)	Pre	28.70 ± 2.80	23.42 ± 1.74	25.69 ± 2.27	26.46 ± 1.63	24.75 ± 1.89	26.79 ± 1.88^a^	31.59 ± 2.25	26.72 ± 1.51^a^	33.19 ± 1.43	29.05 ± 2.73
	Post	27.54 ± 2.16	22.96 ± 1.53	25.13 ± 2.51	24.85 ± 1.58	26.44 ± 1.94	24.57 ± 1.85^b^	31.39 ± 2.22	25.85 ± 1.49^b^	33.58 ± 0.93	28.97 ± 2.20
PLT (×10^3^/μL)	Pre	208.6 ± 28.9^a^	234.3 ± 32.2^a^	147.1 ± 20.4	205.7 ± 30.2	246.3 ± 26	227.8 ± 18.9	275.9 ± 10.9^a^	271.3 ± 21.7	262.9 ± 20.2^a^	234.5 ± 27.1
	Post	126.1 ± 17.2^b^	186.4 ± 21^b^	138.3 ± 18.4	197.9 ± 36.7	216.7 ± 23.2	235.4 ± 24.7	252.4 ± 14.1^b^	265.1 ± 19.5	229.1 ± 22.7^b^	196.8 ± 20.3

Means with different superscript (A, B, C) within rows vary significantly (p < 0.05). Means with different superscript (a, b, c) within columns vary significantly (p < 0.05). *i*-IHD=Incremental intermittent hemodialysis, SE=Standard error, TLC=Total leukocyte count, Hb=Hemoglobin, PCV=Packed cell volume, PLT=Platelet count

### Biochemical analysis

#### Renal function parameters

In both membrane groups, significant reductions in the post-dialysis levels of BUN, creatinine, and phosphorous were consistent throughout the five dialysis sessions (Table-3). A significant reduction in post-dialysis potassium and chloride was observed during sessions I and II in the high-flux group and during sessions III and IV in the low-flux group.

**Table-3:** Pre- and post-*i*-IHD renal function findings (Mean ± SE) of dogs in high-flux (n = 15) and low-flux (n = 15) groups.

Parameter	Dialysis	Session I		Session II		Session III		Session IV		Session V	
				
		High-flux	Low-flux	High-flux	Low-flux	High-flux	Low-flux	High-flux	Low-flux	High-flux	Low-flux
BUN (mg/dL)	Pre	224.5 ± 10.3^a^	206 ± 13.4^a^	155.6 ± 11.3^a^	173.7 ± 10.5^a^	108.9 ± 10.2^a^	137.07 ± 8.48^a^	82.67 ± 5.69^Aa^	132.47 ± 8.18^Ba^	92.93 ± 9.05^a^	103.27 ± 5.81^a^
	Post	120.47 ± 6.51^b^	120.8 ± 7.65^b^	48.4 ± 4.52^b^	60.07 ± 7.27^b^	24.13 ± 4.05^b^	33.4 ± 4.23^b^	15.80 ± 1.47^b^	22.20 ± 1.71^b^	16.33 ± 5.27^b^	10.86 ± 0.99^b^
Creatinine (mg/dL)	Pre	14.04 ± 1.09^a^	14.43 ± 1.47^a^	8.96 ± 0.79^a^	11.03 ± 1.16^a^	9.23 ± 1.06^a^	10.15 ± 1.06^a^	6.77 ± 0.62^a^	7.70 ± 0.59^a^	6.11 ± 0.61^a^	6.90 ± 0.95^a^
	Post	7.113 ± 0.78^b^	8.45 ± 1.01^b^	4.24 ± 0.48^b^	4.99 ± 0.77^b^	2.840 ± 0.46^b^	3.11 ± 0.42^b^	1.80 ± 0.35^b^	2.00 ± 0.22^b^	1.40 ± 0.16^b^	1.98 ± 0.47^b^
Sodium (mEq/L)	Pre	140.27 ± 2.06	142.6 ± 0.82^a^	135.6 ± 2.41	141.07 ± 1.05^a^	134.93 ± 2.03	140.80 ± 0.78	139.93 ± 1.48^A^	141.40 ± 0.51^B^	143.20 ± 1.55^a^	140.27 ± 2.58
	Post	136.33 ± 2.12	138.53 ± 0.65^b^	130.67 ± 2.2	138.67 ± 0.83^b^	135.00 ± 2.17^A^	138 ± 0.61^B^	139.07 ± 0.62	140.00 ± 0.65	139.33 ± 2.19^b^	137.73 ± 1.83
Potassium (mEq/L)	Pre	4.8 ± 0.22^a^	4.793 ± 0.19	4.12 ± 0.28	4.31 ± 0.18^a^	4.93 ± 0.33	4.51 ± 0.18^a^	4.57 ± 0.17	4.70 ± 0.17^a^	4.37 ± 0.30^a^	4.11 ± 0.18
	Post	3.993 ± 0.28^b^	4.527 ± 0.36	3.62 ± 0.25	3.85 ± 0.22^b^	4.25 ± 0.22	3.84 ± 0.19^b^	4.56 ± 0.14	4.10 ± 0.16^b^	3.84 ± 0.25^b^	3.74 ± 0.22
Chloride (mEq/L)	Pre	106.4 ± 1.8^a^	104.47 ± 0.77	102.2 ± 1.37^a^	102.4 ± 1.04	101.27 ± 1.37	102.67 ± 0.82^a^	101.13 ± 0.60	102.33 ± 0.71^a^	104.67 ± 1.70	102.73 ± 2.21
	Post	101.93 ± 1.4^b^	102.73 ± 0.96	97.73 ± 1.1^b^	100.93 ± 0.94	101.87 ± 1.91	99.40 ± 0.82^b^	101.13 ± 0.83	100.80 ± 0.67^b^	105.07 ± 2.40	103.47 ± 1.73
Phosphorous (mg/dL)	Pre	19.49 ± 2.54^a^	18.57 ± 1.83^a^	14.65 ± 1.76^a^	12.21 ± 1.26^a^	13.77 ± 1.39^a^	12.51 ± 1.26^a^	10.03 ± 0.94^a^	12.54 ± 1.27^a^	11.18 ± 1.74^a^	10.85 ± 1.53^a^
	Post	9.4 ± 0.88^b^	9.547 ± 0.99^b^	6.72 ± 0.56^b^	5.8 ± 0.56^b^	5.38 ± 0.45^b^	6.03 ± 0.84^b^	4.84 ± 0.33^b^	6.04 ± 0.45^b^	5.11 ± 0.27^b^	5.72 ± 0.34^b^
Ca (mg/dL)	Pre	11.62 ± 0.62	11.027 ± 0.50	11.74 ± 0.39	10.91 ± 0.35	11.62 ± 0.36	11.36 ± 0.38^a^	11.60 ± 0.35	11.14 ± 0.14	11.00 ± 0.26^a^	10.80 ± 0.18
	Post	10.973 ± 0.32	10.5 ± 0.45	11.14 ± 0.26	10.56 ± 0.29	11.17 ± 0.21	10.66 ± 0.27^b^	11.20 ± 0.15	11.15 ± 0.13	10.21 ± 0.53^b^	10.02 ± 0.66

Means with different superscript (A, B, C) within rows vary significantly (p < 0.05). Means with different superscript (a, b, c) within columns vary significantly (p < 0.05). *i*-IHD=Incremental intermittent hemodialysis, SE=Standard error, BUN = Blood urea nitrogen

#### Liver function parameters

During the session, I, post-dialysis, cholesterol levels were significantly higher in the low-flux group (Table-4). There was a significant reduction and increase in the levels of albumin and triglycerides, respectively, within the low-flux group during post-dialysis, along with a significant increase in the levels of post-dialysis cholesterol in the low-flux group compared with the high-flux group during session II. A significant reduction in post-dialysis TP levels was observed in the low-flux group during session III and in the high-flux group during session V.

**Table-4:** Pre- and post-*i*-IHD liver function findings (Mean ± SE) of dogs in high-flux (n = 15) and low-flux (n = 15) groups.

Parameter	Dialysis	Session I		Session II		Session III		Session IV		Session V	
				
		High-flux	Low-flux	High-flux	Low-flux	High-flux	Low-flux	High-flux	Low-flux	High-flux	Low-flux
Bilirubin (mg/dL)	Pre	0.32 ± 0.11	0.16 ± 0.01	0.21 ± 0.04	0.26 ± 0.1	0.28 ± 0.10	0.36 ± 0.21	0.19 ± 0.01^a^	0.26 ± 0.12	0.18 ± 0.01	0.19 ± 0.01
	Post	0.23 ± 0.03	0.36 ± 0.21	0.22 ± 0.05	0.42 ± 0.26	0.24 ± 0.07	0.28 ± 0.15	0.14 ± 0.01^b^	0.29 ± 0.13	0.17 ± 0.01	0.16 ± 0.01
AST (U/L)	Pre	52.93 ± 7.03^a^	68.1 ± 29.7	67.6 ± 10.1	66.1 ± 18.5	44.27 ± 7.27	79.1 ± 26.9	50.07 ± 5.96	63 ± 16.1	50.07 ± 8.49	61.40 ± 8.66
	Post	66.0 ± 10.8^b^	43.93 ± 3.68	64.8 ± 8.1	58.4 ± 11.3	49.2 ± 6.2	65.4 ± 16.1	52.87 ± 8.98	61.7 ± 13.6	46.67 ± 4.32	70.5 ± 10.5
ALT (U/L)	Pre	65.6 ± 11.4	43.27 ± 4.71	63.3 ± 11.5	49.87 ± 5.4	52.73 ± 6.98	61.9 ± 10.4	47.53 ± 4.22	67.3 ± 12.3	54.07 ± 6.12	64.40 ± 9.62
	Post	66.6 ± 12.3	48.93 ± 4.15	62.7 ± 11.1	51.6 ± 4.35	52.4 ± 7.96	60 ± 10.7	42.60 ± 3.66	63.5 ± 11.8	47.40 ± 5.26	63.9 ± 11.5
ALKP (U/L)	Pre	149.3 ± 31.8	118.2 ± 16.4	130.7 ± 26.7	144.9 ± 21.7	177.2 ± 36.8	153.7 ± 40.1	120.2 ± 18	167 ± 47.9	130.3 ± 35.0	168.1 ± 36.6
	Post	135.3 ± 24.8	126.5 ± 16.7	135.3 ± 26.7	146 ± 20.1	159.9 ± 31.6	172.1 ± 57.9	108.7 ± 16.7	144.1 ± 32.9	121.3 ± 29.6	169.5 ± 30.7
TP (g/dL)	Pre	6.99 ± 0.27	6.64 ± 0.23	6.81 ± 0.29	6.69 ± 0.34	6.52 ± 0.22	6.62 ± 0.28^a^	6.48 ± 0.15	6.58 ± 0.23	7.05 ± 0.16^a^	6.61 ± 0.18
	Post	6.86 ± 0.31	6.76 ± 0.39	6.52 ± 0.26	6.6 ± 0.23	6.41 ± 0.2	6.38 ± 0.25^b^	6.32 ± 0.13	6.48 ± 0.18	6.82 ± 0.17^b^	6.52 ± 0.14
Albumin (g/dL)	Pre	2.95 ± 0.097	2.72 ± 0.12	2.86 ± 0.14	2.74 ± 0.07^a^	2.76 ± 0.10	2.77 ± 0.11^a^	2.84 ± 0.08	2.75 ± 0.06	3.04 ± 0.08	2.96 ± 0.12
	Post	2.88 ± 0.11	2.61 ± 0.11	2.76 ± 0.13	2.54 ± 0.07^b^	2.61 ± 0.10	2.64 ± 0.11^b^	2.71 ± 0.09	2.64 ± 0.06	2.92 ± 0.11	2.79 ± 0.01
GGT (U/L)	Pre	15.00 ± 2.92	9.20 ± 0.87	11.33 ± 2.55	9.40 ± 0.96	9.67 ± 1.20	12.93 ± 3.19	9.33 ± 1.21	8.66 ± 0.88	8.73 ± 1.13	1.27 ± 1.28
	Post	16.53 ± 5.45	8.13 ± 0.79	12.2 ± 2.7	8.4 ± 1.19	8.46 ± 0.96	12.87 ± 3.21	7.86 ± 0.85	7.73 ± 0.47	8.60 ± 0.91	9.40 ± 1.29
Glucose (mg/dL)	Pre	124.27 ± 6.93	112 ± 4.45	117.2 ± 4.13	105.8 ± 5.36	118.27 ± 6.78	104.93 ± 4.67	115.27 ± 5.02	99.13 ± 5.97	111.93 ± 5.92	117.4 ± 8.47
	Post	108.67 ± 6.87	107.93 ± 4.98	123.73 ± 5.05	105 ± 12.1	121.9 ± 11.2	106.07 ± 4.90	105.13 ± 4.63	99.13 ± 3.51	105.47 ± 4.39	112.73 ± 3.59
Triglyceride (mg/dL)	Pre	80.67 ± 5.46	77.47 ± 4.92	80.4 ± 5.47	70.33 ± 4.71^a^	79.53 ± 2.81	77.2 ± 4.1	78.13 ± 4.74	75.93 ± 3.86	84.07 ± 5.12	78.47 ± 4.87
	Post	86.13 ± 4.26	85.13 ± 3.50	81.73 ± 4.61	84.93 ± 4.64^b^	75.8 ± 3.27	88.27 ± 4.53	80.40 ± 3.20	87.27 ± 6.71	84.4 ± 4.71	88.73 ± 7.40
Cholesterol (mg/dL)	Pre	193.73 ± 7.05	209.6 ± 7.36	210.07 ± 6.46	197.87 ± 9.02	209.47 ± 8.96	205.00 ± 6.11	197.47 ± 7.49	193.47 ± 6.96	196.47 ± 8.09	195.20 ± 8.42
	Post	188.8 ± 5.1^A^	218.5 ± 10^B^	198.4 ± 7.66^A^	199.4 ± 7.6^B^	205.33 ± 9.08	205.9 ± 10.1	200.00 ± 7.48	199.1 ± 10.2	200.27 ± 8.33	194.80 ± 7.53

Means with different superscript (A, B, C) within rows vary significantly (p < 0.05). Means with different superscript (a, b, c) within columns vary significantly (p < 0.05). *i*-IHD=Incremental intermittent hemodialysis, SE=Standard error, ALT=Alanine transaminase, AST=Aspartate aminotransferase, GGT=Gamma-glutamyl transferase, ALKP=Alkaline phosphatase, TP=Total protein

### *i*-IHD and dialysis adequacy

The mean ± SE URR, KT/v, and CrRR of Groups I and II from sessions I to V did not show any significant differences between the groups during each session (Table-5).

**Table-5:** Dialysis adequacy based on comparison of URR, KT/v and CrRR (Mean ± SE) of dogs in high-flux (n = 15) and low-flux (n = 15) groups.

*i*-IHD	URR		KT/v		CrRR	
		
	High-flux	Low-flux	High-flux	Low-flux	High-flux	Low-flux
Session I	46.09 ± 2.07	41.38 ± 1.65	0.66 ± 0.04	0.68 ± 0.12	48.65 ± 4.73	42.15 ± 3.22
Session II	68.73 ± 2.08	66.25 ± 2.72	1.42 ± 0.08	1.26 ± 0.09	52.31 ± 4.76	55.93 ± 3.20
Session III	78.85 ± 1.88	76.32 ± 2.02	1.94 ± 0.11	1.77 ± 0.10	69.04 ± 2.87	69.27 ± 2.55
Session IV	83.23 ± 0.70	80.51 ± 1.52	2.05 ± 0.12	2.18 ± 0.06	73.49 ± 3	73.53 ± 2.29
Session V	83.81 ± 2.70	89.46 ± 0.72	2.48 ± 0.17	2.94 ± 0.25	76.04 ± 2.19	73.07 ± 2.19

*i*-IHD=Incremental intermittent hemodialysis, SE=Standard error, URR=Urea reduction ratio, KT/v=Fractional urea clearance, CrRR=Creatinine reduction ration

### QOL*i*

The mean ± SE QOL*i* scores in the high- and low-flux membrane groups from day 0 (pre-dialysis) to day 45 post-dialysis are presented in Table-6. Scores ranged from 1 to 5, with 1 denoting the lowest score and 5 denoting the highest score. On day 0, the quality of life of the client was significantly higher in the low-flux group than in the high-flux group. On day 15, hygiene was scored higher in the high-flux group, whereas mobility was scored higher in the low-flux group. On day 30, dogs in the high-flux group showed significantly better appetite, whereas dogs in the low-flux group had significantly better hygiene and general health. On day 45, appetite was better in the low-flux group, whereas mobility and general health were better in the high-flux group. The QOL*i* within the high- and low-flux membrane group using Kruscal–Wallis test from day 0 to day 45 showed a significant increase in scores of happiness, mental status, mobility, appetite, general health, overall quality of life assessment, and pain in the high-flux group (Table-7) whereas, in the low-flux group, a significant increasing trend in scores of mental status, appetite, hygiene, water balance, mobility, general health, overall quality of life assessment, and pain was noted. The QOL*i* scores of the clients also showed a steep increase from day 0 to day 45 compared with the low-flux membrane group.

**Table-6:** QOL*i* score (Mean ± SE) of dogs in high-flux (n = 15) and low-flux (n = 15) groups from day 0 pre-dialysis to day 45 post-dialysis by Mann-Whitney test.

QOL*i* domain	Membrane type	Day 0			Day 15			Day 30			Day 45		
			
		Mean ± SE	W*	p-value	Mean ± SE	W*	p-value	Mean ± SE	W*	p-value	Mean ± SE	W*	p-value
Happiness	High-flux	1.73 ± 0.118	210.5	0.338	2.75 ± 0.140	240.5	0.746	4.53 ± 0.063	247.5	0.470	4.57 ± 0.051	217.5	0.470
	Low-flux	1.62 ± 0.064			2.82 ± 0.156			4.57 ± 0.051			4.53 ± 0.063		
Mental status	High-flux	1.48 ± 0.044	243	0.643	2.62 ± 0.055	245.5	0.534	3.08 ± 0.039	219.5	0.456	2.91 ± 0.060	241.0	0.707
	Low-flux	1.62 ± 0.107			2.66 ± 0.046			3.06 ± 0.048			2.93 ± 0.048		
Appetite	High-flux	1.24 ± 0.068	233.5	0.698	2.64 ± 0.060	244.5	0.538	3.33 ± 0.072	161.0	0.001	3.31 ± 0.039	317.5	0.000
	Low-flux	1.24 ± 0.094			2.66 ± 0.046			3.04 ± 0.044			3.60 ± 0.048		
Hygiene	High-flux	1.73 ± 0.048	225	0.645	2.33 ± 0.032	155.5	0.000	2.28 ± 0.091	309.5	0.000	2.33 ± 0.032	225.0	0.695
	Low-flux	1.69 ± 0.039			2.08 ± 0.039			2.64 ± 0.022			2.26 ± 0.081		
Water balance	High-flux	1.56 ± 0.045	232.5	1.000	2.03 ± 0.033	262.5	0.078	2.60 ± 0.053	219.0	0.438	2.66 ± 0.063	210.0t	0.213
	Low-flux	1.57 ± 0.045			2.16 ± 0.063			2.53 ± 0.059			2.56 ± 0.045		
Mobility	High-flux	2.00 ± 0.032	217.5	0.439	2.60 ± 0.035	339.0	0.00	3.22 ± 0.062	248.0	0.373	3.68 ± 0.051	126.0	0.000
	Low-flux	1.91 ± 0.076			3.00 ± 0.032			3.31 ± 0.039			3.22 ± 0.062		
General health	High-flux	1.56 ± 0.137	221	0.630	2.96 ± 0.033	225.0	0.577	3.50 ± 0.048	344.0	0.000	4.50 ± 0.048	120.5	0.000
	Low-flux	1.47 ± 0.103			2.93 ± 0.045			4.43 ± 0.045			3.50 ± 0.048		
QOL assessment	High-flux	1.53 ± 0.133	210	0.288	3.66 ± 0.159	230.5	0.936	4.13 ± 0.090	218.5	0.343	4.13 ± 0.090	225.0	0.577
	Low-flux	1.33 ± 0.126			3.60 ± 0.190			4.00 ± 0.109			4.06 ± 0.066		
QOL of owner	High-flux	3.20 ± 0.058	289	0.016	4.15 ± 0.077	253.0	0.390	4.46 ± 0.010	247.5	0.533	4.55 ± 0.036	229.5	0.914
	Low-flux	3.39 ± 0.058			4.27 ± 0.061			4.54 ± 0.037			4.51 ± 0.097		
Pain	High-flux	1.17 ± 0.117	259	0.197	1.44 ± 0.062	263.5	0.138	1.51 ± 0.055	208.0	0.231	1.62 ± 0.030	232.5	1.000
	Low-flux	1.24 ± 0.082			1.53 ± 0.054			1.44 ± 0.062			1.62 ± 0.030		

QOL*i*=Quality-of-life index, SE=Standard error

**Table-7:** QOL*i* score (Mean ± SE) of dogs in high-flux (n = 15) and low-flux (n = 15) groups from day 0 pre-dialysis to day 45 post-dialysis by Kriscal-wallis test.

QOL*i* domain	High-Flux				Low-Flux			
	
	Day 0	Day 15	Day 30	Day 45	Day 0	Day 15	Day 30	Day 45
Happiness	1.73 ± 0.118^A^	2.75 ± 0.140^A^	4.53 ± 0.063^B^	4.57 ± 0.051^B^	1.62 ± 0.064^A^	2.82 ± 0.156^A^	4.58 ± 0.051^B^	4.53 ± 0.063^B^
Mental status	1.48 ± 0.044^A^	2.62 ± 0.055^B^	3.08 ± 0.039^C^	2.91 ± 0.060^BC^	1.62 ± 0.010^AB^	2.67 ± 0.046^AB^	3.07 ± 0.048^C^	2.93 ± 0.048^C^
Appetite	1.24 ± 0.068^A^	2.64 ± 0.060^A^	3.33 ± 0.072^B^	3.31 ± 0.039^B^	1.24 ± 0.094^A^	2.67 ± 0.046^AB^	3.04 ± 0.044^C^	3.60 ± 0.048^AB^
Hygiene	1.73 ± 0.048^A^	2.33 ± 0.032^B^	2.28 ± 0.091^B^	2.33 ± 0.032^B^	1.68 ± 0.039^A^	2.08 ± 0.039^AB^	2.64 ± 0.022^C^	2.26 ± 0.081^B^
Water balance	1.56 ± 0.045^A^	2.03 ± 0.033^B^	2.60 ± 0.053^C^	2.66 ± 0.063^C^	1.56 ± 0.045^A^	2.16 ± 0.630^B^	2.53 ± 0.059^C^	2.56 ± 0.054^C^
Mobility	2.00 ± 0.032^A^	2.60 ± 0.035^B^	3.22 ± 0.062^C^	3.68 ± 0.051^C^	1.91 ± 0.076^A^	3.00 ± 0.032^B^	3.31 ± 0.039^B^	3.22 ± 0.062^B^
General health	1.56 ± 0.137^A^	2.96 ± 0.033^AB^	3.50 ± 0.048^B^	4.50 ± 0.048^C^	1.46 ± 0.103^A^	2.93 ± 0.045^AB^	4.43 ± 0.045^C^	3.50 ± 0.048^B^
QOL assessment	1.53 ± 0.133^A^	3.66 ± 0.159^B^	4.13 ± 0.090^C^	4.13 ± 0.090^C^	1.33 ± 0.126^A^	3.60 ± 0.190^B^	4.00 ± 0.097^BC^	4.06 ± 0.066^BC^
QOL of owner	3.20 ± 0.058^A^	4.15 ± 0.076^B^	4.46 ± 0.102^C^	4.55 ± 0.036^C^	3.39 ± 0.058^A^	4.27 ± 0.061^B^	4.54 ± 0.037^B^	4.51 ± 0.097^B^
Pain	1.17 ± 0.117^A^	1.44 ± 0.062^A^	1.51 ± 0.055^B^	1.62 ± 0.030^B^	1.24 ± 0.082^A^	1.53 ± 0.054^AB^	1.44 ± 0.062^AB^	1.62 ± 0.030^B^

Means with different superscript (A, B, C) within rows vary significantly (p < 0.05). QOL*i*=Quality-of-life index, SE=Standard error

## Discussion

In the present study, the majority of dogs with Stage IV were above 6 years of age. Similar to our results, a previous study by Supriya [10] reported that the age at presentation of dogs with renal failure ranged from 4 to 8 years. The average body weight of dogs was above 25 kg, which may be because all dogs in our study were large breeds (Golden Retrievers, Labrador Retrievers, and German Shepherds). The preference of urban residents to keep pets as a status symbol, as well as due to pet affection, can be a reason for more urban representation in our study.

Proteinuria, a risk factor for the progression of CKD, was a consistent finding in urine examination that may be attributed to glomerular or tubular lesions, but glomerular lesions were more likely as they resulted in a greater magnitude of proteinuria (2+). The reduced kidney size on X-ray examination may be attributed to the loss of nephrons due to the progression of kidney disease. Lobacz *et al*. [11] reported that a kidney length of 2.5–3.5 times with respect to L_2_ vertebrate is valid. The ultrasound findings of this study are more common in later stages (III and IV) of CKD and are often considered indicative of irreversible kidney damage [12]. Increased cortical echogenicity may be due to fibrosis, sclerosis, or infiltration, whereas loss of corticomedullary differentiation may be associated with inflammatory diseases, such as glomerulonephritis, interstitial nephritis, and pyelonephritis [13].

The exact mechanism of CKD-induced hypertension is not fully understood, but an amalgamation of arteriolar scarring and glomerular capillary hampered renal vasodilation prostaglandins, activation of renin-angiotensin system in response to impaired sodium clearance, and exorbitant renin secretion are commonly implicated [14]. Increased production of angiotensin II and aldosterone is a result of increased renin secretion. Angiotensin II, apart from its pressor effect on the sympathetic nervous system, which results in CKD, also causes vasoconstriction of the efferent arterioles, thus aggravating intraglomerular hypertension. Furthermore, tissue remodeling and kidney fibrosis may also be attributed to angiotensin and aldosterone. For hypertension management (SBP >160 mmHg), dogs in both groups were administered either monotherapy or combination therapy on day 0. In our study, the significant reduction in SBP in both membrane groups can be attributed to the antihypertensive treatment protocol with ACEI and CCB. The mechanism by which ACEI (enalapril) might reduce SBP is by dilating efferent arterioles, resulting in a net reduction of internal pressure. Similarly, CCB (amlodipine), carries a weaker central chronotropic and inotropic effect, leading to a direct vasodilatory effect on the peripheral vasculature [14]. A previous study by Komeno *et al*. [15] has suggested hypotension as the most serious complication during IHD in dogs, and it is mostly attributed to loss of blood volume in the extracorporeal circuit at the beginning of dialysis along with activation of inflammatory mediators when blood comes in contact with the tubing and dialyzer membrane. No such episode of intra-dialytic hypotension was observed in our study, and it may be attributed to pediatric blood tubing with a priming volume of 117 mL compared to adult tubing with a priming volume of 172 mL. Further, in our study, there was a significant reduction in the post-dialysis SBP as compared with pre-dialysis SBP in both groups, and a significant reduction in SBP was achieved after 5 *i*-IHD sessions with no significant difference between the two groups. No study on the effects of two different membranes (i.e., high- and low-flux membranes) has previously been undertaken in veterinary medicine. However, only a few recent studies in human patients are available [16, 17].

Multiple factors may contribute to reduced Hb levels after dialysis, with a major loss of some volume of blood in the extracorporeal circuit [18]. Apart from these, anemia is a common presentation in dogs with CKD, and the hampered reduction of erythropoietin by the diseased kidneys is its major cause [19]. In dogs undergoing *i*-IHD, leukocytosis and absolute neutrophilia after a dialysis session may be due to the direct contact of blood with the dialysis membranes and extracorporeal circuit blood tubing, which may elicit a series of changes in blood cells. A significant reduction in the post-dialysis PLT may be attributed to anticoagulation therapy with heparin sodium during dialysis to prevent blood from clotting in the extracorporeal circuit. As a frequently observed hematological alteration in patients undergoing hemodialysis, this thrombocytopenia may also be due to the activation of platelets with adhesion and complement activation, irrespective of membrane. It is estimated that there is approximately a 15% drop in the PLT during hemodialysis, which recovers hours after the dialysis [20].

BUN, creatinine, and phosphorous are the most common uremic parameters used to monitor the efficacy of dialysis sessions [21]. The failure of kidneys to excrete toxins leads to their retention in the body and is considered a major contributor to uremia in dogs with CKD. These uremic compounds are classically grouped into three major categories depending on their molecular weight and protein binding ability, that is, small solutes (<500 Dalton) with a high degree of water solubility and low or no protein binding (BUN, creatinine, phosphorous, sodium, potassium, water, phosphate, calcium, etc.); middle solutes (500–60,000 Dalton) with good protein binding ability (β2 microglobulin, parathyroid hormone, carbamylated, protein, granulocyte inhibitory proteins, and other peptides); and protein-bound uremic toxins. As such, dialyzers can be divided into two main types depending on their pore size and β2 microglobulin clearance over 20 mL/min [22], that is, high- and low-flux. Recent studies on the adequacy of high-flux versus low-flux membranes [22, 23] in human patients have indicated the diffusion of greater amounts of metabolic toxins, including middle-sized molecules, by using high-flux membranes compared to low-flux membranes. In our study, dialysis outcomes involving both high- and low-flux membranes in canine patients showed no significant differences in the clearance of both membranes over a period of five dialysis sessions. However, during session IV, the high-flux membrane group showed significantly lower pre-dialysis BUN and sodium levels compared to the low-flux membrane group, which may be attributed to variable dietary intake. It is important to note that the pre-dialysis levels of BUN and creatinine were higher in both high- and low-flux groups and showed significant post-dialysis reduction, but their levels again increased in subsequent pre-dialysis samplings, indicating the need for additional modifications during non-dialysis days like renal diet, omega-3 and 6 fatty acids, L-carnitine, gut prebiotics and probiotics and anti-fibrotics. A similar short-term reduction in uremic toxins was documented in a previous study by Momeni *et al*. [21] involving dogs, but no additional nutritional strategies were employed during non-dialysis days to counter this repeated increase in short intervals. Phosphorous levels also showed a significant reduction in both membrane groups in our study. After each dialysis session, dogs in both groups were prescribed an oral phosphate binder, that is, calcium acetate with meals along with a renal prescription diet low in phosphorous content. Calcium acetate binds to the phosphorous present in the meal before being absorbed in the gastro-intestinal tract (GIT). Studies in patients undergoing dialysis [24] using the one-dose/one-meal balanced theory have shown it to be more potent in reducing phosphorous levels than calcium carbonate or calcium citrate.

Although dogs in the present study had triglycerides and cholesterol levels within the normal physiologic range, reduced levels of triglycerides and cholesterol were observed consistently in the high-flux group (post-dialysis) compared with the low-flux group. The observed reductions in triglycerides and cholesterol levels after dialysis with high-flux membranes may be attributed to the effects on the recovery of lipoprotein lipase activity [25]. A recent study on the effect of high-and low-flux membranes on cardiac risk factors in human children with end-stage renal disease reported a statistically significant difference (reduction) in total cholesterol, high-density lipoprotein-cholesterol and triglyceride levels after 6 months in the high-flux membrane group [26]. The findings of the present study in veterinary medicine hold significance because cardiovascular diseases (hypertension and left ventricular hypertrophy) are major concerns in dogs with stage IV CKD. As such, dogs diagnosed with cardiovascular abnormalities along with CKD may be preferred for high-flux membrane dialysis given that this study demonstrated that such dogs may benefit in some markers of dyslipidemia (triglycerides and cholesterol) using high-flux membranes. There was a significant reduction in TP and albumin during the second and third dialysis sessions (post-dialysis) in the low-flux group and a significantly lower TP during 5^th^ session (post-dialysis) in the high-flux group. It is understood that dialysis itself is a major cause of nutrient loss into dialysate, thus necessitating extra supplementation of amino acids and vitamins in patients undergoing hemodialysis.

In this study, the 3-weekly IHD model was modified to incorporate the concept of *i*-IHD. Initially, we performed three IHD sessions on alternate days (thrice weekly model) to achieve patient stabilization. After that, dialysis was performed after an increment of 15 days to evaluate whether initial patient stabilization can keep the dog going without dialysis for 15 days. To maintain the dog for an extended period of time (15 days), between subsequent dialysis sessions, we incorporated renal-specific nutritional support (omega-3 and 6-fatty acids, L-carnitine, gut pre-pro-biotics, and anti-fibrotic renal supplement) along with other medications (anti-hypertensives, proton-pump inhibitor, furosemide, sodium bicarbonate, and phosphate binder). Dialysis sessions were conducted according to previous recommendations, in which treatment intensity prescription was developed depending on the session (1^st^, 2^nd^, 3^rd^, 4^th^, and 5^th^) and pre-dialysis BUN concentration [9]. In order to avoid any life-threatening dialysis complication (dialysis disequilibrium syndrome and hypotension), we fixed our URR target of 40%–50%, 60%–70%, 70%–80%, and >80% along with KT/v targets of 0.60–0.80, 0.81–1.50, 1.51–2.0 > and >2.0 during sessions I, II, III, IV, and V, respectively. In contrast to veterinary medicine, human medicine broadly classifies dialyzer adequacy into three groups based on KT/v and URR, that is, inadequate dialysis (KT/v ≤0.89; URR ≤0.60), relatively adequate dialysis (KT/v = 0.90–1.29, URR = 0.61–0.70) and totally adequate dialysis (KT/v ≥1.3, URR ≥0.70) [23]. Such a contrast may be attributed to dogs’ and cats’ relatively lower body weight than humans (less body weight = less blood volume). No studies on dialysis adequacy involving high- and low-flux membranes have been published in veterinary medicine, even after thorough searches using various available scientific journal search engines (Google, Cera, PubMed). However, recent studies involving human patients have reported better dialysis adequacy with high-flux membranes than with low-flux membranes [23, 27]. Although our results demonstrated no significant difference in the clearance rates of both high- and low-flux membranes, the clearance of triglyceride and cholesterol was better in the high-flux group, suggesting these membranes’ potential in dogs with concurrent cardiovascular involvement. CrRR was calculated as per the formula provided by Cowgill [9] and ranged from 42% to 76% from session I through session V. No significant difference was observed in CrRR between the membrane groups. Creatinine levels decreased during each dialysis session as the volume of blood processed also increased from session I through session V, leading to more pronounced creatinine clearance as the sessions progressed.

Our QOL*i* contained 32 items under 10 domains, and it benefitted from its ease of completion and simple wording. On average, clients took around 10 min to complete the form, which prevented a lack of interest by the clients. Previously, pet-oriented QOL surveys were more complex and contained >35 items, resulting in a lack of interest in completing the questionnaire [28]. Likewise, another QOL for canines in Europe contained 109 items within 13 domains, and the study concluded that with such a large number of items, respondents’ attention is expected to decline as the survey time increases, thus deviating from its reliability [29]. Although more improvement in QOL*i* for dogs undergoing IHD is required, this preliminary study demonstrates that simply asking the client some simple questions like “How is your dog after a previous dialysis session?” reassures the client that the veterinarian shows concern about their pet’s health and considers its wellbeing to be paramount. Apart from this, we also benefitted from QOL*i* in the way that it helped us adjust the dog’s medications according to the QOL*i* assessment. As an example, if one client reported that his dog was eating very less between dialysis sessions, then we included appetite stimulants containing buclizine at 1 mL/5kg orally twice a day (Syrup Aptiquik, Mankind Pharma, India), which improved the pet’s appetite in subsequent QOL*i* assessments. Thus, a concise and reliable QOL*i* for the evaluation of dogs with CKD who are undergoing *i*-IHD provided us with excellent guidance for determining what is more important to the dog and the steps that should be taken to take care of such concerns. It also gave a sense of involvement to the client in their pet’s health and treatment, which led to more critical thinking and observation on the part of client while answering the survey. In fact, we observed that over time, on repeated filling-out of QOL*i* questionnaires, client became more particular, and they did monitor most of the changes in their pets in relation to the QOL*i* domains. Similar findings were reported by earlier researchers who documented that QOL surveys influence therapy decisions and treatment responses [27, 29].

## Conclusion

To the best of our knowledge, this is the first study in the world to investigate the potential of *i-*IHD in dogs with CKD involving high- and low-flux membranes. Furthermore, no such study has been conducted in veterinary medicine in which a detailed dialysis-related QOL*i* has been evaluated over time in dogs with CKD. The present study demonstrated that dogs suffering from CKD can be efficiently managed with *i-*IHD by undergoing the first three dialysis sessions on alternate days, followed by an increment of 15 days. It was demonstrated that a high-flux membrane can significantly reduce triglyceride and cholesterol levels compared with a low-flux membrane, making a high-flux membrane a suitable option for dogs with concurrent cardiovascular involvement and undergoing dialysis. Overall, no significant difference was observed between both membranes’ clearance rates (dialysis adequacy). Our study also demonstrated that a dialysis-related QOL*i* can be very helpful in making clinical decisions along with instilling a sense of involvement in the client’s mind regarding their pet’s health and treatment. The authors propose that limiting the QOL*i* questionnaire and extending the study period could enhance this research on the average survival timespan of *i*-IHD dogs in CKD.

## Data Availability

All data generated or analyzed during the study are included in this article.

## Authors’ Contributions

RS and DKG: Conceived and designed the study. AS and RS: Performed dialysis and QOL*i* assessment. RSS: Analyzed and interpreted the data. RS and RSS: Drafted and revised the manuscripts. All authors have read, reviewed, and approved the final manuscript.
